# Research on farmers’ adoption of additional technology combinations: Practice from Chongqing, China

**DOI:** 10.1371/journal.pone.0294862

**Published:** 2023-11-30

**Authors:** Yu Li, Zhiheng Zhang

**Affiliations:** 1 College of Economics and Management, Southwest University, Chongqing, 400715, China; 2 School of Accounting, Chongqing University of Technology, Chongqing, 400054, China; West Bengal State University, INDIA

## Abstract

Tobacco farmers often adopt additional multiple agricultural technologies (AMATs) in addition to implementing the standardized technical system in China. Based on the cross-sectional micro data of 346 households of Chongqing, China, this paper assesses the determinants and impacts of the adoption of AMATs on income by using a multinomial endogenous treatment effects model to correct for selection bias and endogeneity caused by observed and unobserved heterogeneity. The results show that (1) the adoption of combinations of AMATs is determined by the household head’s education level, experience in tobacco growing, the shortest distance to nearby town, the amount of technical training, the ratio of land available for mechanical cultivation to tobacco land, the distance to extension station, and the ratio of leased land. (2) The adoption of combinations of AMATs has heterogeneous effects on farmers’ income through yield and quality improvement. (3) The comprehensive combination of AMATs is not necessarily the best option for farmers. Due to the interaction between technologies such as complementary, substitute or supplementary effects, the moderate implementation of fertilizers and soil improvement is the most effective combination. The results of this research provide a scientific basis for improving the adoption efficiency of AMATs in China.

## Section 1: Introduction

Modern agricultural technologies are widely acknowledged to increase agricultural productivity, combat climate change, guarantee food security and reduce poverty [1–6]. For a long time, public and private extension agents have paid attention to promoting all kinds of advanced agricultural technologies to farmers. However, the technology adoption of farmers in many developing economies is not satisfactory and is often characterized by inadequate or excessive adoption [7]. On the one hand, some farmers still have low adoption rates of some beneficial technologies [8,9], which restricts income growth. On the other hand, some farmers tend to over-adopt technologies, which lead to the low efficiency of production input. In particular, the excessive input of some chemical elements, such as pesticides and fertilizers, causes serious environmental pollution and harm to farmers’ health [10–14].

When faced with various technologies in the process of agricultural production, farmers can adopt them either individually or in combination [5,15,16]. Feder [17] first attempted to evaluate the interaction of various agricultural technologies adopted by farmers. Recently, studies on the adoption of multiple agricultural technologies (MATs) in the joint estimation framework have begun to increase [1,18,19]. Some scholars have considered conservation agriculture (CA), climate-smart agriculture (CSA), integrated soil fertility management (ISFM), sustainable agricultural practices (SAPs), and sustainable intensification practices (SIPs) as research objects to analyze the factors of various technologies adopted by farmers [4,9,20–22]. Some studies have further analyzed the impact of different technology combinations on outcomes [2,5,6,23,24]. These studies have drawn some different conclusions on the relationship between the comprehensiveness of adopted technology combinations and outcomes.

According to some scholars, the adoption of technology combinations brings about greater benefits than the adoption of individual-use technology. As the complexity of the adopted technology portfolio increases, so does the income (output) effect. Yang et al. [25] found that a combination of straw returning, subsoiling and organic manure is the best method for rice production in China. Other researchers [3,19,26–29] have found similar results in studies of the adoption of combinations of MATs. However, some studies have shown that the adoption of technology combinations that are more comprehensive is not always better. Adolwa et al. [8] conducted research in Northern Ghana and Western Kenya and found that while farmers’ adoption of ISFM can increase corn production, the yield and household income do not increase with the increase in technology components. Other studies also suggest that it is not the most comprehensive technology combination that maximizes family benefits [5,24,27,30,31]. Moreover, Zeweld et al. [29] reported that Northern Ethiopia farmers’ combined usage of animal manure and retention of crop residues generates negative effects. Therefore, it is necessary to analyze the effectiveness of different combinations of adopting MATs based on paying continuous attention to the factors related to adopting MATs. For a large number of farmers with limited resource endowment in developing economies, it is valuable to adopt reasonable technology combinations to improve both technology input‒output efficiency and outcomes.

In China, contract farming operations are a typical production organization model used to realize the organic connection between small farmers and modern agricultural development [32]. To ensure the yield and quality of scattered farmers, companies often develop mandatory standardized technical systems. However, as the entities of production and operation, farmers have certain autonomy in adopting technology. Our long-term observations show that Chinese farmers commonly adopt additional multiple agricultural technologies (AMATs) as a supplement to standardized technical systems to maximize their yields and/or incomes due to plot heterogeneity. The intensive usage of modern elements is generally recognized as an important process of agricultural development in low-income countries [10,33]. The combination of input technologies such as fertilizers, pesticides, and herbicides can significantly increase yields [11,12,34]. However, the situation is quite different in China, which is the world’s largest consumer of agricultural chemicals [35]. Due to inefficient usage and a high loss ratio, China experiences a series of negative effects on food safety, the environment and human health [34–38]. Although China implemented the “Zero Growth Action Plan of Chemical Fertilizer and Pesticide Use” from 2015 to 2020 [39], the current usage level is still higher than the average level globally, and the issue of how to reduce the quantity and increase efficiency is still a long-term and difficult task.

Chongqing is located in southwest China and belongs to the upper reaches of the Yangtze River. The area is mainly hilly and mountainous, with fragmented and less arable land. Moreover, the arable land is almost always sloping more than 25 degrees and facing certain problems, such as intensifying soil pollution, declining land fertility and serious soil erosion. To promote the sustainable development of the Yangtze River Economic Belt and reduce the pollution caused by fertilizers and pesticides, it is necessary to strengthen the investigation and evaluation of AMATs adoption in Chongqing.

Studies on the adoption of MATs have begun to increase, and most empirical studies have been conducted in less-developed economies such as Africa and Central America. It is rare to analyze the technology adoption behavior of tobacco farmers in Chongqing, China [25,40]. This study contributes to the empirical literature in three ways. (1) The research object of this paper is a variety of technology adoption behaviors located outside the standardized technical system; previous studies have focused on the standardized technical system normally promoted by agricultural technology extension agents, without considering the AMATs located outside the standardized technical system adopted by tobacco farmers under contract farming. (2) This paper reveals the heterogeneous impact of the adoption of different AMAT combinations on income, which helps improve the input‒output efficiency of technologies. Due to the complementarity, substitution or supplementary effects found among various technologies, the additional adoption of different combinations has a complex impact on income. Selecting an appropriate technology combination can effectively improve the efficiency of technological output and reduce negative environmental externalities. (3) Considering the heterogeneity of agricultural product quality, this paper analyzes the impact of different AMAT combinations on yield growth and quality improvement. Previous studies have generally assumed that agricultural products are homogeneous and that technological progress affects tobacco farmers’ income mainly by improving agricultural productivity. However, in reality, advanced agricultural technologies can improve not only yields but also the quality of agricultural products by affecting the growth process. For cash crops, the prices of different quality grades are often very different, which has a significant impact on farmers’ income.

The results of this paper help to improve the input‒output efficiency of tobacco farmers’ technology adoption from the perspective of additional technology combinations beyond standardized production. It has important policy significance in promoting the effective diffusion of agricultural innovations and reducing the negative externalities of the environment. Although this paper focuses on Chongqing, China, our empirical findings can also be applied to other regions of China. It is of certain reference significance to a large number of developing economies that have transformed from self-sufficient traditional agriculture to commercial agriculture.

The remainder of the paper is organized as follows. Section 2: Theoretical analysis framework; Section 3: Data source, model setting, and variable selection; Section 4: Results; Section 5: Discussion; Section 6: Conclusions and implications.

## Section 2: Theoretical analysis framework

The adoption of agricultural technologies depends on maximizing the expected utility or benefits to the farmer, which often needs to be balanced among the economy, the environment, risk and other factors. Feder et al. [15] analyzed the factors of adoption of new agricultural technologies, including access to credit, access to technical information, risk aversion, incentives, land tenure, human capital investment, farm size, and labor constraints. The existing studies have shown that the factors of different technology adoption decisions are different in different space-time conditions and natural environments [29,41–43]. Ruzzante et al. [44] summarized the general function of technology adoption as follows:

Adoption=f(X) (1)

where “adoption” is the observed adoption behavior of the farmer, and is a matrix of socioeconomic, personality, environmental, farm financial, farm management, or external factors, which are usually gathered through surveys. In general, these factors have an impact on all technology adoption behavior of farmers, which further affects agricultural outcomes.

In developing countries, contract farming has played an important role in improving the technology adoption level of small farmers. Contract farming refers to an organizational form of agricultural cooperation based on contracts signed between farmers and enterprises. Before agricultural production, legally binding contracts are signed between farmers and enterprises. Based on the contract, farmers organize production, and enterprises purchase products produced by farmers. In order to ensure that the quality and quantity of products produced by farmers meet the standards, companies generally promote a set of standardized technical systems on a mandatory basis, thus, farmers’ adoption behavior is passive to a certain extent. However, the adoption of AMATs is the “active” behavior of farmers’ completely independent choice. There are some differences between the two mechanisms. The company’s promotion of a standardized technical system is based on regional commonness and normal climatic conditions. The standardized technical system often designs various technical input standards based on the average soil fertility level. However, due to the heterogeneity of plot conditions, there are differences in soil nutrient demand [45], and the unified factor input standard stipulated by standardized production aggravates the mismatch between fertilizer application and nutrient demand. Therefore, it is necessary to adopt AMATs for farmers. Based on the assumption of rational economic man, the adoption of AMATs by farmers produces additional expenses such as learning costs, information search costs and production factor costs. Although these costs increase, the adoption of AMATs often occurs in practice. This reflects that the common requirements of a standardized technical system have difficulty suiting plot characteristics and the resource endowment of each farmer. When adopting AMATs, farmers need to take into consideration the amount of input cost, whether the expected goal is achieved, and whether the risk of technology adoption is controllable. Thus, such adoption is also the result of multiple factors interacting.

Based on Ruzzante et al. [44], this paper divides the factors of farmers’ adoption of AMATs into farmer/farm household characteristics, farm biophysical characteristics, financial characteristics, external factors, and “perception” variables. (1) Farmer/farm household characteristics. The differences in household heads’ individual and family endowments will lead to different views on the same technology and the adoption of different behaviors [15]. The existing studies mainly study the influence of farmer/farm characteristics on farmers’ technology adoption behavior from the variables of gender [1], age [19], education level [46] and household size [28]. (2) Farm biophysical characteristics. Cultivated land is the material condition of agriculture, and the status of farmland resources owned by farmers has an important impact on technology adoption. Previous studies have focused on the influence of the biophysical characteristics of farms on farmers’ technology adoption behavior from the aspects of land size [23] and land quality [9]. (3) Financial characteristics. Technology adoption requires capital and factor investment; therefore, financial characteristics play an important role in technology adoption. The existing studies mainly research the impact of financial characteristics on technology adoption from market distance [31], access to credit [23], land tenure [20], livestock ownership [3], off-farm income [26]. (4) External factors. Technology adoption decisions depend on farmers’ understanding of new technologies; those who do not understand new technologies generally cannot adopt new technologies [47]. Access to technology extension or information support, technology diffusion among farmers [9,21], and participation in cooperatives and other farmer organizations generally have positive effects on farmers’ technology adoption [27]. (5) Farmers’ “perception” variables. With the deepening of the research, some scholars explain the adoption behavior of farmers from the perspective of farmers’ ideas and attitudes and other psychological factors due to the limited explanatory power of the above factors. Some studies also use “perception” variables [1], such as perception of soil fertility level [22] and perception of risk [48].

Under contract farming, tobacco farmers usually adopt AMATs for contingency management and soil conservation beyond the implementation of standardized technical systems. Contingency management refers to the occurrence of abnormal and irregular changes in the production process, which requires tobacco farmers to take immediate management measures or preventive production measures. Cultivated land is an important carrier of human social and economic activities. Because tobacco farmers’ plots are scattered, soil conditions vary greatly, and soil conservation technologies often need to be implemented continuously over the years. Therefore, it is generally not included in the company’s standardized technical system. To improve the conditions for continuous cultivation and increase land productivity, some tobacco farmers will adopt soil conservation technology independently. Tobacco farmers in Chongqing generally adopt additional pesticides, additional fertilizer and soil improvement for contingency management and soil conservation in addition to standardized technical systems. Depending on the different combinations of the three AMATs, there are seven possible packages that tobacco farmers can adopt: (1) P_1_F_0_S_0_, (2) P_0_F_1_S_0_, (3) P_0_F_0_S_1_, (4) P_1_F_1_S_0_, (5) P_1_F_0_S_1_, (6) P_0_F_1_S_1_, and (7) P_1_F_1_S_1_. P, F and S refer to additional pesticide, additional fertilizer, and soil improvement, respectively. The subscripts “1” and “0” denote adoption and nonadoption, respectively.

The existing studies have proven that different technologies are not independent of each other, and there are generally complementary, substitutive or supplementary effects among the technologies adopted by farmers [20,22,26,43,49]. Amadu et al. [1] and Canales et al. [50] also showed that complementarities among technical combinations can improve the benefits. Correspondingly, the reason why adopting multiple technologies at the same time is not as good as partial adoption may be that there is a substitution effect among related technologies such that each technology influences others produce adverse effects. In addition, due to regional differences in climate, soil conditions and crop types, the adoption of different combinations of MATs will have differential impacts on productivity and livelihoods [51]. Therefore, farmers are faced with either adopting two or more technologies at the same time or including multiple components of the complex technology system in the specific ecological environment, such as CA, ISFM, CSA, and SAPs. It is necessary to distinguish the possible complementarity, substitution or supplementary effects between technologies and weigh the input‒output differences of different technology combinations comprehensively. This paper analyzes Chongqing tobacco farmers’ seven alternatives involving the three AMATs to identify the key factors that affect decisions to adopt different technology combinations and help tobacco farmers choose the optimal package and achieve the best allocation of resources, which not only maximizes outcomes but also considers environmental sustainability.

Based on the analysis above, this paper proposes the following hypotheses:

### Hypothesis 1

There is significant heterogeneity in the influence of various factors on tobacco farmers’ adoption of AMAT combinations.

### Hypothesis 2

Tobacco farmers are more likely to adopt additional combinations of technologies rather than a single technology due to the effects of complementarity, substitution or supplementation between technologies affecting the input‒output efficiency of additional technology combinations.

### Hypothesis 3

The comprehensiveness of the additional technology combinations adopted by tobacco farmers is convex due to the excessively comprehensive technology combinations reducing the input‒output efficiency.

## Section 3: Data source, model setting, and variable selection

### Data source

Tobacco is an important cash crop that is under quota control in China (According to The World Health Organization Framework Convention on Tobacco Control, China government makes commitment to strengthen tobacco control.), and its production adopts a typical contract farming operations model. Chongqing is one of the major tobacco-producing areas in China, with a cultivated farm size of approximately 400 thousand mu (1 mu = 1/15 hectares). The Chongqing Tobacco Company (CTC) began to establish the ISO9000 quality management system for tobacco planting in 2008 and implemented the Good Agricultural Practices (GAPs) in 2012. Every year, considering various influencing factors, the tobacco company will formulate a set of standardized technical systems for seedling raising, mechanical tillage, plant protection, baking, grading and other production processes that serves as a mandatory technical specification for tobacco farmers to implement. On the basis of adopting a standardized technical system, tobacco farmers often adopt additional pesticides, additional fertilizer, and soil improvement.

The current survey was conducted in the main tobacco-growing areas of Chongqing, including Fengjie, Wuxi, Wushan, Fengdu, Wulong, Qianjiang, Pengshui, Youyang, and Shizhu. The cultivated farm size of tobacco in each county is over 25 thousand mu. To ensure the representativeness of the research data and the reliability and validity of the survey, our research adopted a stratified and multistage probability sampling method and selected 5% of tobacco farmers as the sample objects. We distributed the sample quantity of each district and county in proportion according to the planned quantity of tobacco production in nine tobacco-growing districts and counties in 2018, for a total of 465 questionnaires. Firstly, nine major production districts and counties are selected as the primary sampling units. Secondly, sort and number the production of tobacco planting townships from small to large in each district and county, and determine 30 sample townships based on the random number results. Thirdly, number all tobacco farmers in every sampled townships, and determine 465 sample tobacco farmers based on the random number results. Finally, conduct a one-on-one household survey on these sample farmers. The field survey was conducted from March to June in 2019.

Similar to tobacco farmers in Kenya [52] and Malawi [53], tobacco farmers in Chongqing are mainly engaged in tobacco production, with few other employment opportunities and income sources. Referring to Suri [54], household labor costs are not included in the tobacco planting costs, and the total income function is used to approximate the income function. The survey did not collect the production cost information of tobacco farmers. Li et al. [55] took a similar approach.

In addition, we obtained the real data of tobacco farmers selling tobacco to CTC in 2018 by cooperating with the Chongqing Tobacco Science Research Institute, which is a subsidiary of the Chongqing Tobacco Company. We matched the sample questionnaire with the real data one by one and excluded the samples with incomplete data to ensure the objective reality of the empirical data. Finally, 346 valid questionnaires were obtained, with an effective rate of 74.41%.

### Model setting

Referring to Varma [56], this paper analyzes the adoption of AMATs based on the random utility framework. Tobacco farmers will adopt a combination that can maximize their utility *U*_*ij*_ by comparing various combinations of AMATs subject to various constraints. Accordingly, if *U*_*ij*_>*U*_*ik*_, *k*≠*j*, then an *i*th tobacco farmer will choose combination *j* over any alternative combination *k*.

When making technology adoption decisions, farmers may be affected by observable and unobservable factors related to outcome variables, resulting in endogeneity problems [57]. Referring to Manda et al. [24], Varma [56], and Yang et al. [25], we applied the multinomial endogenous treatment effects (METE) model proposed by Deb and Trivedi [58] to analyze the factors and impacts of the adoption of different combinations of AMATs. The METE model allows the use of latent factor structures to specify the distributions of endogenous treatments (using AMATs) and outputs (various welfare variables), allowing a distinction between selection on observables and selection on unobservables [58]. METE model considers both the interdependence of decision-making and the selection bias caused by observed and unobserved features. There are two stages in the application of METE model. Firstly, to model the behaviour of decision-adopting based on the mixed multinomial logit selection model. Secondly, to measure the impact of AMATs combinations on outcome variables based on OLS with selectivity correction.

The METE model consists of two stages. In the first stage, tobacco farmer *i* chooses one of the seven AMAT combinations. Following Deb and Trivedi [58,59], let *U*_ij_* denote the indirect utility obtained by tobacco farmer *i* from selecting the *j*th AMAT combination, *j* = 0, 1, 2… as follows:

$$U_{ij}{}^{*}=z_{i}^{'}\alpha_{j}+\sum_{k=1}^{J}\delta_{jk}l_{ik}+n_{ij}$$ (2)

where *z*_*i*_ denotes the vector of farmer/farm household characteristics, farm biophysical characteristics, financial external characteristics, and farmers’ “perception” variables with associated parameters *α*_*j*_; *n*_*ij*_ represents i.i.d. error terms; and *l*_*ik*_ is a latent factor that incorporates the unobserved characteristics common to tobacco farmer *i*‘s treatment choice and outcome variables. Following Deb and Trivedi [58], let *j* = 0 denote the control or base group and *U*_*i*0_* = 0 = 0. Although *U*_ij_* is not observed, let *d*_*j*_ be the observable binary variables representing the choice of combinations of AMATs and serving as a vector of d_*i*_ = (*d*_*i*1_,*d*_*i*2_,…,*d*_*iJ*_). Similarly, let l_*i*_ = (*l*_*i*1_,*l*_*i*2_,…,*l*_*iJ*_); then, the probability of treatment can be represented as follows:

$$\mathrm{Pr}\left(d_{i}|z_{i},l_{i}\right)=g(z_{i}^{'}\alpha_{1}+\sum_{k=1}^{J}\delta_{1k}l_{ik}+z_{i}^{'}\alpha_{2}+\sum_{k=1}^{J}\delta_{2k}l_{ik}+\cdots +z_{i}^{'}\alpha_{J}+\sum_{k=1}^{J}\delta_{Jk}l_{ik})$$ (3)

As recommended by Deb and Trivedi [58], we assume that g has a mixed multinomial logit (MMNL) structure, which is defined as follows:

$$\mathrm{Pr}(d_{i}{|z}_{i},l_{i})=\frac{\exp(z_{i}^{'}\alpha_{j}+\delta_{j}l_{ij})}{1+\sum_{k=1}^{J}\exp(z_{i}^{'}\alpha_{k}+\delta_{k}l_{ik})}$$ (4)

The analysis of the effect of adoption of AMAT combinations on yield, price, and income (the natural logarithms) is undertaken in the second stage. The expected result equation formula is as follows:

$$E(y_{i}{|d}_{i},x_{i}{,l}_{i})=x_{i}^{'}\beta +\sum_{j=1}^{J}r_{j}d_{ij}+\sum_{j=1}^{J}\lambda_{j}l_{ij}$$ (5)

where *y*_*i*_ represents the outcome variables of yield, price and income for tobacco farmer *i*, whereas x_*i*_ is a set of exogenous variables with associated parameter vectors *β*, and *r*_*j*_ denotes the treatment effects relative to the control group, i.e., nonadopters of AMATs. The latent factor is *l*_*ij*_, indicating that the outcome variable may be influenced by unobserved characteristics that also affect the choice of combinations. When the factor loading *λ*_*j*_ is positive (negative), treatment and outcome are positively (negatively) associated with unobserved variables. This implies that there is positive (negative) selection. Because the outcome variables—yield, price and income—are continuous variables, we assume that they follow a normal distribution function. The model was estimated using the maximum simulated likelihood (MSL) approach (command “mtreatreg” in Stata 14).

Following Deb and Trivedi [58], the METE model is often identified by the inclusion of instrumental variables (IVs). Valid instrumental variables will have an impact on the adoption decisions of technology combinations but are hardly expected to affect outcomes such as tobacco farmers’ yield and income. Referring to Gao et al. [40] and Khonje et al. [46], we used the leased land ratio and the distance from tobacco farmer households to the nearest technology extension station as our instrumental variables.

The leased land ratio reflects the impact of land ownership (or land tenure) on technology adoption. Ownership of land ensures long-term cash flow, and tobacco farmers working on their own land are more likely to adopt technology [49]. However, the lack of land ownership can prevent tenants from benefiting from future technological gains because of the risk of eviction. There is an inevitable need to lease land for tobacco farmers in Chongqing to realize large-scale operation. Therefore, the leased land ratio was chosen as the proxy of the impact of land ownership on the adoption of AMAT combinations. Tobacco technicians provide useful information on advanced agricultural technologies; therefore, the shorter distance to the extension station could help tobacco farmers adopt AMAT combinations. The two instrumental variables passed this simple falsification test, which suggests that they jointly affect the adoption decisions of the AMATs but do not affect the outcome variables (Tables 1 and 2).

**Table 1:** Mixed multinomial logit model estimates of adoption of AMATs.

Variables	P_1_F_0_S_0_	P_0_F_1_S_0_	P_1_F_1_S_0_	P_0_F_0_S_1_	P_1_F_0_S_1_	P_0_F_1_S_1_
Age	-0.117	0.0886	0.0232	0.0554	0.0393	0.0145
	(0.132)	(0.126)	(0.0304)	(0.0824)	(0.0655)	(0.0268)
Education level	-0.911	0.964	0.0648	-1.940**	0.0798	-0.477*
	(1.202)	(1.278)	(0.289)	(0.873)	(0.731)	(0.261)
Farming experience	0.00509	-0.184	-0.00485	-0.0466	0.0158	-0.0858***
	(0.0808)	(0.127)	(0.0230)	(0.0593)	(0.0577)	(0.0203)
Home size	-2.156*	2.024*	0.256	0.0927	-0.342	-0.0224
	(1.135)	(1.202)	(0.159)	(0.413)	(0.332)	(0.160)
Tobacco farming labor	2.064	0.000396	0.431	0.637	-0.720	0.320
	(1.354)	(1.258)	(0.303)	(1.018)	(0.983)	(0.288)
Farm size	0.0375	0.00923	0.0161	0.0455*	0.0126	0.00995
	(0.0317)	(0.0674)	(0.0106)	(0.0266)	(0.0282)	(0.0101)
Distance to township	-0.107	0.218**	0.0799***	-0.0820	-0.0825	-0.0630
	(0.126)	(0.109)	(0.0253)	(0.135)	(0.104)	(0.0402)
Loan	-0.697	-0.814	-0.234	-0.167	-0.916	-0.000290
	(1.545)	(1.889)	(0.433)	(1.222)	(1.111)	(0.378)
Number of trainings	-0.0296	0.0738	0.0920	0.344*	-0.455**	0.240***
	(0.218)	(0.437)	(0.0767)	(0.203)	(0.226)	(0.0698)
Labor force ratio	-4.474*	13.59	-0.0199	-2.161	-3.336*	0.628
	(2.546)	(9.393)	(1.027)	(2.628)	(1.858)	(0.959)
Flat land ratio	-4.608*	1.162	-1.227*	-2.235	-2.653**	-1.597***
	(2.436)	(4.787)	(0.640)	(1.818)	(1.348)	(0.583)
Instrumental variables						
Distance to extension station	0.120	-0.264	-0.108**	-0.443*	-0.186	-0.250***
	(0.121)	(0.345)	(0.0503)	(0.241)	(0.200)	(0.0654)
Leased land ratio	0.286	-6.557*	-0.552	0.340	0.418	-1.607***
	(1.072)	(3.607)	(0.709)	(0.681)	(0.435)	(0.592)
Constant	12.73	-29.57**	-5.264**	-1.955	3.715	1.698
	(10.49)	(14.91)	(2.131)	(6.249)	(4.578)	(2.026)

Joint significance of instrumental variables *χ*^2^ (12) 32.06**.

Wald test *χ*^2^ = 107.61; P > *χ*^2^ = 0.0148.

Note: The reference category is P_1_F_1_S_1_.

***, **, and * denote statistical significance at the 1%, 5%, and 10% levels, respectively. Standard errors in parentheses.

**Table 2:** The F test is used to verify the validity of the instrumental variables.

Variables	P_1_F_1_S_0_	P_0_F_1_S_1_	P_1_F_1_S_1_
Ln yield	F(2, 22) = 0. 45	F(2, 48) = 1.60	F(2, 212) = 1.31
Ln price	F(2, 22) = 1.17	F(2, 48) = 1.79	F(2,212) = 0.30
Ln income	F(2, 22) = 0.84	F(2, 48) = 1.15	F(2,212) = 2.10
No. of obs.	36	62	226

As shown in Table 2, under the three combinations of P_1_F_1_S_0_, P_0_F_1_S_1_ and P_1_F_1_S_1_, the F test of the instrumental variables on yield, price and income is not significant.

### Selection of variables

Broad literature has studied the factors influencing farmers’ technology adoption [9,15,42,44,60]. Knowler and Bradshaw [41] found that only a few variables can generally explain the adoption of CA in various studies. Zeweld et al. [29] pointed out that the impact of the same variable is different in the adoption of different agricultural practices. Based on the previous theoretical analysis, we selected farmer/household characteristics (household head of gender, age, education level, tobacco farming experience, and home size, tobacco-growing labor force), farm biophysical characteristics (arable land size), financial characteristics (loan, distance to township), external factors (number of trainings), and “perception” variables. Furthermore, we also considered the following variables in combination with the reality of Chongqing.

Labor force ratio: Farmers’ decision to adopt technology is affected by the status of the family labor force. This variable is used to reflect the proportion of labor (16–65 years old) owned by tobacco farmers’ households to all family members.

Village cadre: Social capital plays an important role in the diffusion of agricultural innovations [31]. Considering that village cadres usually have wider social networks and easier access to technical information, we chose whether there is a village cadre in the household as a proxy for social capital.

The flat land ratio: Farmers’ decision to adopt technology is also affected by the slope of the plot [3,24]. We used the flat land ratio as a proxy for slope. The proportion of land available for mechanical cultivation to tobacco land reflects these convenient conditions for tobacco farmers to adopt mechanization technology. The higher the index is, the more inclined tobacco farmers are to choose mechanized production, which can reduce costs and improve productivity. Tobacco planting lands in Chongqing are mainly distributed in mountainous areas; the CTC has carried out large-scale land consolidation to promote agricultural machinery in recent years. The increase in the proportion of flat area is not only conducive to standardized production but also conducive to improving the efficiency of AMAT combinations.

The definitions of all variables used in the empirical analysis are presented in Table 3.

**Table 3:** Variable definition, measurement and descriptive statistics.

Variables	Delimiting and measuring	Mean
Outcome variables		
Yield	Yield per mu in kilograms	114.40 (30.53)
Price^a^	Selling price (yuan^b^) per kilogram	27.90 (2.05)
Income	Per-capita income for household laborers engaged in tobacco growing(10000 Yuan)	5.97 (3.92)
Treatment variables		
Additional pesticide	1 if additional pesticide is adopted; 0 otherwise	0.79 (0. 41)
Additional fertilizer	1 if additional fertilizer is adopted; 0 otherwise	0. 95 (0.22)
Soil improvement	1 if soil improvement is adopted; 0 otherwise	0.87 (0. 34)
Explanatory variables		
Gender	Gender of the household head (1 = male, 0 = female)	0. 94 (0. 24)
Age	Age of the household head (years)	50.11 (7.22)
Education level ^c^	Standardized education received by household head	2.62 (0. 72)
Farming experience	Tobacco farming experience of household head (years)	21.10 (9.69)
Home size	Number of all the family members in the household	4.93 (1.43)
Social capital	1 if at least one village cadre in the household; 0 otherwise	0.15 (0. 36)
Tobacco farming labor	Number of tobacco farming labor in the household	2.08 (0. 60)
Farm size	Cultivated farm size of tobacco (mu)	37.70 (21.06)
Distance to township	The shortest distance to the towns nearby(km)	8.23 (7.05)
Loan	Need taking a loan from bank (1 = Yes)	0. 45 (0. 50)
Number of trainings	Number of technical trainings in 2018	5.26 (2.60)
Labor force ratio	Labor force (16–65 years old) as a proportion of total households	0. 69 (0. 24)
Flat land ratio	Land suitable for mechanical farming as a percentage of total tobacco land	0. 71 (0. 31)
Instrumental variables		
Distance to extension station	The shortest distance to technology extension station nearby(km)	5.25 (4.57)
Leased land ratio	The percentage of leased land in all cultivated land	0. 67 (0. 51)
No. of obs.		346

Note

^a^ The price is the average sales price of tobacco leaves, which is equal to the total sales amount of tobacco farmers divided by the total sales volume, reflecting the quality level of tobacco leaves.

^b^ The exchange rate at the time of the survey was approximately 1 USD = 6.64 yuan.

^c^ Numbers from 1 to 5 indicate no schooling, primary school, junior high school, senior middle school, college and above, respectively. Standard errors in parentheses.

## Section 4: Results

### Characteristics of tobacco farmers adopting AMATs

Table 3 provides the descriptive statistics of the relevant variables. It shows that, on average, household heads are over 50 years old, male (94%), and have less than a junior high school education level. Most of them are professional farmers who have grown tobacco for a long time, with more than 21 years of planting experience on average. In 2018, they attended technical training 5.26 times on average. The sample families have 4.93 members, the labor force ratio is 0.69, and the cultivated farm size of tobacco is approximately 37.70 mu (the flat land ratio is 0.71, and the leased land ratio is 0.67). The average tobacco yield is 114.40 kg/mu. The average price is 27.90 yuan/kg. The average income is 59.7 thousand yuan. On average, it is 8.23 kilometers from the nearest township and 5.25 kilometers from the nearest technology extension station. Fifteen percent of the sample families have village cadres, indicating that their social capital is good. Less than half (45%) of tobacco farmers need loan to produce their crop. Under the support of the existing financial environment and loan policies, they can obtain bank loan smoothly, which shows that tobacco farmers have sufficient capital guarantees for their production.

By analyzing the sample information, it can be found that only one tobacco farmer did not adopt any additional pesticides, additional fertilizer, or soil improvement. Therefore, this observation sample was excluded. The empirical study used 345 samples. From the distribution of technology portfolios (Table 4), it can be seen that most of the samples selected the combination of P_1_F_1_S_1_, which accounted for 65.51%, 10.43% chose P_1_F_1_S_0_, and 17.97% chose P_0_F_1_S_1_. We also find that the number of other combination samples is very small. Therefore, the follow-up of this research will focus on P_1_F_1_S_1_, P_1_F_1_S_0_ and P_0_F_1_S_1_. The statistical characteristics of P_1_F_1_S_1_, P_1_F_1_S_0_ and P_0_F_1_S_1_ are listed in Table 5 below.

**Table 4:** Proportion of farmers adopting different technology combinations.

	P_1_F_0_S_0_	P_0_F_1_S_0_	P_1_F_1_S_0_	P_0_F_0_S_1_	P_1_F_0_S_1_	P_0_F_1_S_1_	P_1_F_1_S_1_	Total
Freq.	4	4	36	6	7	62	226	345
Percent	1.04	1.04	10.43	1.57	2.03	17.97	65.51	100.00

**Table 5:** Descriptive statistics of P_1_F_1_S_0_, P_0_F_1_S_1_, P_1_F_1_S_1_.

Variables	P_1_F_1_S_0_		P_0_F_1_S_1_		P_1_F_1_S_1_	
Yield		117.167		111.373		113.000
		(2.701)		(2.862)		(1.851)
Price		27.024		28.559		27.840
		(0.354)		(0.195)		(0.139)
Income		6.194		5.616		6.044
		(0.654)		(0.439)		(0.278)
Gender		0.889		0.919		0.942
		(0.053)		(0.035)		(0.016)
Age		50.528		49.645		49.947
		(1.248)		(0.868)		(0.475)
Education level		2.722		2.516		2.659
		(0.130)		(0.079)		(0.049)
Farming experience		22.944		16.145		22.075
		(1.810)		(1.009)		(0.637)
Home size		5.389		4.742		4.920
		(0.274)		(0.161)		(0.095)
Social capital		0.139		0.113		0.159
		(0.058)		(0.041)		(0.024)
Tobacco farming labor		2.250		2.129		2.044
		(0.122)		(0.093)		(0.034)
Farm size		41.472		35.516		37.896
		(3.893)		(2.385)		(1.433)
Distance to township		11.753		5.677		8.462
		(1.753)		(0.546)		(0.448)
Loan		0.444		0.468		0.442
		(0.084)		(0.064)		(0.033)
Number of trainings		5.333		6.323		4.982
		(0.481)		(0.307)		(0.166)
Labor force ratio		0.676		0.713		0.695
		(0.037)		(0.029)		(0.016)
Flat land ratio		0.653		0.639		0.749
		(0.050)		(0.043)		(0.019)
Distance to extension station		5.5		3.148		5.888
		(0.641)		(0.340)		(0.329)
Leased land ratio		0.638		0.597		0.690
		(0.053)		(0.036)		(0.040)
No. of obs.		36		62		226

### Factors of the adoption of AMATs

We observe that more than 65% of tobacco growers use all three AMATs, and there are no samples that do not use AMATs (Table 4). Inspired by Chiputwa et al. [61], who analyzed and compared the impacts of three sustainability-oriented standards on the livelihoods of smallholder coffee farmers in Uganda using propensity score matching with multiple treatments for the empirical analysis regarding no adoption as the baseline and any of three schemes as the baseline for each other, we adopt a combination of three AMATs as the reference category. This differs from the common practice of using nonadopters as a control group [24,46,56]. Table 1 shows the parameter estimates of the MMNL model, which is equivalent to the first stage of the METE model. The model fits the data very well according the Wald test, *χ*^2^ = 107.61; P > *χ*^2^ = 0.0148, implying that the null hypothesis that all the regression coefficients are jointly equal to zero can be rejected. The results indicate that the estimated coefficients substantially differ across alternative combinations of AMATs.

The empirical results show that, on average, the flat land ratio and the distance to extension station have significant statistical significance in regard to P_1_F_1_S_0_, P_0_F_1_S_1_, and P_1_F_1_S_1_. The coefficient of the flat land ratio on P_1_F_1_S_0_ is -1.227 at the 10% significance level, and the coefficient on P_0_F_1_S_1_ is -1.597 at the 1% significance level, which shows that this variable has a more significant negative effect on P_0_F_1_S_1_. The coefficient of the distance to extension station on P_1_F_1_S_0_ is -0.108 at the 5% significance level, and the coefficient on P_0_F_1_S_1_ is -0.250 at the 1% significance level, which indicates that this variable has a more significant negative effect on P_0_F_1_S_1_.

Three variables, the education level of the household head, farming experience and leased land ratio, have significant negative impacts on P_0_F_1_S_1_. The coefficient of the education level of the household head is -0.477 at the 10% significance level. The coefficient of farming experience is -0.0858 at the 1% significance level. The coefficient of the leased land ratio is -1.607 at the 1% significance level. The number of trainings has a significant positive effect on P_0_F_1_S_1_, and the coefficient is 0.240 at the 1% significance level.

In addition, distance to township has a positive effect on P_1_F_1_S_0_, and the coefficient is 0.0799 at the 1% significance level.

The empirical results also show that neither the age of household heads, home size, tobacco farming labor, cultivated farm size, loan nor labor force ratio are significant. It should be noted that when the model regression considers the iterated concave, the gender of the household head and whether there is a village cadre are removed.

### Average treatment effects of different combinations of AMATs

Table 6 shows the results of estimates of the impact of adopting AMATs on yields, prices and incomes.

**Table 6:** The METE model estimates of the impacts of AMATs on yields, prices and incomes.

AMATs Package	Ln Yield	Ln Price	Ln Income
**Exogenous**			
P_1_F_1_S_0_	0.149	0.0155	0.218**
	(0.182)	(0.0329)	(0.0972)
P_0_F_1_S_1_	0.0932**	0.0311	0.139*
	(0.0427)	(0.0193)	(0.0762)
**Endogenous**			
P_1_F_1_S_0_	0.165*	-0.00108	0.125***
	(0.0963)	(0.00259)	(0.0292)
P_0_F_1_S_1_	0.0778	0.0663***	0.212***
	(0.103)	(0.00320)	(0.0475)
Age	-0.00353*	-0.00101***	-0.00965***
	(0.00204)	(0.0000888)	(0.000845)
Education level	-0.00662	0.0190***	0.0845***
	(0.0197)	(0.000636)	(0.00976)
Farming experience	-0.000862	0.000108*	0.00356***
	(0.00155)	(0.0000645)	(0.000756)
Home size	0.00412	-0.000597	-0.00256
	(0.0110)	(0.000372)	(0.00496)
Tobacco farming labor	-0.0235	0.0113***	-0.337***
	(0.0230)	(0.000793)	(0.00837)
Farm size	-0.00234***	0.0000125	0.0199***
	(0.000707)	(0.0000319)	(0.000251)
Distance to township	-0.00227	0.000143**	-0.000751
	(0.00195)	(0.0000689)	(0.00132)
Loan	-0.00735	-0.0300***	0.0215*
	(0.0287)	(0.00110)	(0.0119)
Number of trainings	0.00537	0.00382***	0.0163***
	(0.00524)	(0.000179)	(0.00206)
Labor force ratio	-0.0367	-0.00852***	-0.0695**
	(0.0649)	(0.00214)	(0.0317)
Flat land ratio	-0.0463	0.0301***	-0.0426**
	(0.0441)	(0.00139)	(0.0185)
Constant	5.092***	3.269***	1.737***
	(0.147)	(0.00578)	(0.0612)
**Selection terms (λ)**			
P_1_F_1_S_0_	-0.00601	0.0108***	0.103***
	(0.0322)	(0.000527)	(0.00564)
P_0_F_1_S_1_	0.0240	-0.0402***	0.0746***
	(0.0308)	(0.000539)	(0.00791)

Note: The reference category is P_1_F_1_S_1_.

***, a statistical significance at the 1%, 5%, and 10% levels, respectively. Standard errors in parentheses.

The second-stage regression is not discussed to conserve space, but the results are reported in Table 6. For comparison, outcome variables are estimated under the assumptions of exogenous and endogenous adoption decisions of AMATs with P_1_F_1_S_1_ as the reference category.

Under the exogenous assumption, the results show that, on average, P_0_F_1_S_1_ has significant effects on yield and income, while P_1_F_1_S_0_ only has a significant impact on income. It is not difficult to find that after controlling for unobserved heterogeneity, the average adoption effect shows a certain difference (Table 6). P_0_F_1_S_1_ has significant positive impacts on price and income. P_1_F_1_S_0_ has significant positive impacts on yield and income. Compared with the adoption of the combination of the three AMATs, on average, P_0_F_1_S_1_ results in an increase of 6.63% in price and an increase of 21.20% in income. P_1_F_1_S_0_ results in an increase of 16.50% in yield and an increase of 12.50% in income.

The above empirical results show that although the sample tobacco farmers adopt AMATs at a high level, most of them choose P_1_F_1_S_1_, and the average income treatment effect is not as good as that for P_0_F_1_S_1_ or P_1_F_1_S_0_.

## Section 5: Discussion

### Analysis of factors of the adoption of AMATs

Compared with the combination of the three AMATs, the empirical results (Table 1) show that the factors mainly include the education level of household head, tobacco farming experience, distance to township, amount of training, flat land ratio, distance to extension station, and leased land ratio.

The education level of the household head has a significant negative effect on P_0_F_1_S_1_, which indicates that the higher the education level is, the less likely tobacco farmers are to adopt P_0_F_1_S_1_, and the more willing they are to adopt P_1_F_1_S_1_. It may be that tobacco farmers are generally poorly educated; therefore, tobacco farmers with relatively higher education levels can understand the benefits of increasing their technological input and are more willing to adopt the combination of the three AMATs. Similarly, Yang et al. [25] found that the education level of Chinese farmers has a positive effect on the comprehensive combination of soil conservation practices (SCPs).

Farming experience has a very significant negative effect on P_0_F_1_S_1_, indicating that with increasing planting experience, the possibility of adopting P_0_F_1_S_1_ decreases, and tobacco farmers are more willing to adopt P_1_F_1_S_1_. A possible reason is that the sampled farmers are professional tobacco farmers. They have experienced the process from the traditional backward production mode to the extensive application of modern technology and obtained the benefits of agricultural innovation practices. Therefore, they believe that increasing their technological input will help to increase their outcome, and they tend to adopt a more comprehensive combination.

Distance to township has a positive impact on P_1_F_1_S_0_, indicating that the farther away from a township they are, farmers are significantly more likely to adopt P_1_F_1_S_0_ rather than P_1_F_1_S_1_. A reason may be that increased travel time and transaction costs restrict the adoption of the combination of all three AMATs. Mutenje et al. [31] found that distance to market has a negative and significant impact on the adoption of a combination of all three agricultural innovations. Manda et al. [24] also found that the farther away Zambian farmers are from the fertilizer and product market, the less likely they are to adopt SAPs. The results also show that Chongqing tobacco farmers, who are relatively isolated and have few information sources, rely heavily on traditional farming culture and experience and are accustomed to increasing the input of pesticides and fertilizers.

The number of trainings has a strong positive impact on P_0_F_1_S_1_, indicating that with an increase in training times, tobacco farmers have a deeper understanding of technology and thus pay more attention to sustainable development rather than blindly choosing the most comprehensive AMATs. Adopting soil improvement technology will help to fundamentally control some unfavorable factors that affect the growth and development of tobacco leaves and cause soil degradation, which can improve soil properties and soil fertility to create good soil environmental conditions for the sustainable development of tobacco.

The flat land ratio has the most significant positive impact on P_1_F_1_S_1_, followed by P_1_F_1_S_0_ and P_0_F_1_S_1_. This indicates that the larger the flat land proportion is, the more likely tobacco farmers are to adopt P_1_F_1_S_1_ than P_1_F_1_S_0_ or P_0_F_1_S_1_. It may be that the larger the flat land ratio is, the easier it is for tobacco farmers to implement a more comprehensive package. In Chongqing, there are many mountainous areas; however, with the promotion of mechanized land consolidation by the CTC, the flat land ratio increases, and tobacco farmers are willing to adopt a more comprehensive technology portfolio. These conclusions are different from those of Manda et al. [24], who found that the probability of adopting the combination of all the SAPs is lower for plots with gentle slopes.

Distance to extension station has significantly negative effects on P_0_F_1_S_1_ and P_1_F_1_S_0_, indicating that tobacco farmers prefer to adopt P_1_F_1_S_1_ over P_0_F_1_S_1_ or P_1_F_1_S_0_ with increasing distance. It may be that the closer they are to a technology extension station, tobacco farmers are more likely to receive technical training and guidance. Thus, they have a better understanding and perception of technology and will selectively adopt AMATs. Farmers who are far away from those extension stations are not conducive to obtaining technical information, so they have a greater psychological dependence on comprehensive technology combinations and tend to increase their investment in various factors. Therefore, a more comprehensive package of AMATs is preferred.

The leased land ratio has a significant negative impact on P_0_F_1_S_1_. Tobacco farmers with a larger leased land ratio are less likely to adopt P_0_F_1_S_1_ and more likely to adopt P_1_F_1_S_1_. The result is different from that in the study by Manda et al. [24], who found that households that rent part of their land are less likely to adopt SAP packages than households that own their own land. It might be that these households studied sustainable development technologies, which had a long payback time and a large impact on the uncertainty of leased land. Perhaps tobacco farmers who lease more land in Chongqing pay more attention to short-term benefits to compensate for the cost of leased land and are thus more willing to adopt more comprehensive AMATs to maximize their income.

Consistent with Tambo and Mockshell’s study on CA adoption [28], the empirical results show that the age of the household head is not significant, indicating that both young and old tobacco farmers are likely to adopt various combinations of the three AMATs. The possible reasons include poor education among tobacco farmers and the lack of independent technical information about agricultural innovations, both of which lead to a herd mentality when AMATs are adopted.

The results for home size, tobacco farming labor and labor force ratio are also not significant. There may be two reasons for this outcome. On the one hand, the CTC adjusts the production organization mode and deepens the agricultural division of labor when implementing the standardized technical system, and specialized services are provided in multiple production processes. On the other hand, tobacco farmers generally employ short-term workers during the busy season; thus, only a small number of family tobacco laborers can achieve large-scale planting.

The empirical results show that cultivated farm size is not significant, indicating that tobacco farmers with different cultivated farm sizes are also likely to adopt various combinations of the three AMATs. Different from the research of many scholars (e.g., Khonje et al. [30], Makate et al. [23] and Yang et al. [25]), the authors found that the area of cultivated land is often positively related to the adoption of more complex technology combinations. This paper studies the additional technology combination beyond standardized production, and the land cultivated by Chongqing tobacco farmers is generally small. We find that the farm size has no significant difference on the impact of adopting additional pesticide, additional fertilizer and soil improvement.

The voluminous literature on technology adoption shows that access to credit has a significant positive impact on technology adoption decisions [15,23,57]. However, our regression results show that the factor of whether farmers take out loan is not significant; we think this is because Chongqing tobacco farmers may commonly have sufficient funds for the adoption of AMATs and thus do not need to buy technology investment through credit. In addition, even if tobacco farmers are short of liquidity, they can easily obtain special-purpose loan from banks with tobacco cultivation contracts. This finding indicates that Chongqing tobacco farmers are not subject to liquidity constraints in adopting AMATs.

It is worth noting that gender and village cadre were removed in the regression model because we considered there to be iterative concavity. Based on our field research, tobacco farmers are organized to join cooperatives by the CTC, and the agricultural technology extension services to tobacco farmers are inclusive, without differences in gender and social status.

Based on the above analysis, the individual characteristics of the household head, farm biophysical characteristics, financial characteristics and external factors all have different influences on the adoption of AMATs. Thus, Hypothesis 1 has been proven.

### Analysis of the average treatment effects of different combinations of AMATs

With the assumption of the exogenous adoption of AMATs, as seen in Table 6, the results show that the application of P_0_F_1_S_1_ not only increases the yield but also increases income compared with adopting all three AMATs. However, P_1_F_1_S_0_ is only good for boosting income. The above inference may be misleading because it is only based on observed features and ignores the effects of unobserved factors. The difference in outcomes could be caused by unobserved characteristics of the tobacco farmers, such as their innate managerial and technical abilities [57]. To take into account unobserved factors, a METE model is estimated to overcome this issue.

The average effects of adoption after controlling for unobserved heterogeneity show different results with the significant improvement of the validity of estimation. P_0_F_1_S_1_ has no significant effects on yield but a significant positive impact on price and income. P_1_F_1_S_0_ has a significant positive impact on yield and income. P_0_F_1_S_1_ mainly improves the quality of tobacco leaves, with no impact on yields. P_1_F_1_S_0_ obviously promotes the yield but perhaps has a negative effect on the quality of tobacco leaves (the regression result is negative but not significant). What needs special emphasis is that although the adoption of P_1_F_1_S_0_ significantly increases the yield, the CTC can only purchase the quota agreed upon in the contract. Therefore, the results of the income effect show the actual income increase of yield within the quota but cannot reflect the income of the output exceeding the quota. The estimates found after accounting for unobserved factors are relatively high compared to the results found under the exogenous assumption, suggesting that ignoring endogeneity underestimates the actual impact of adoption.

As seen in Table 6, the factor loading (λ) of P_0_F_1_S_1_ in the income equation shows positive selection bias, which indicates that unobserved confounders increasing the likelihood of adopting P_0_F_1_S_1_ are associated with higher levels of income. Negative selection bias is evident in the price equation, which suggests that unobserved variables increasing the likelihood of adopting P_0_F_1_S_1_ are associated with lower levels of price. The factor loadings (λ) of P_1_F_1_S_0_ in the price and income equations all show positive selection bias, which suggests that unobserved factors increasing the likelihood of adopting P_1_F_1_S_0_ are associated with higher levels of price and income.

Combining descriptive statistical results and empirical results of samples, it can be seen that tobacco farmers are more likely to adopt a combination of additional agricultural technologies rather than a single technology. Moreover, because of the interaction between technologies, different combinations have different income effects. Thus, Hypothesis 2 is verified.

In summary, although most of the sampled tobacco farmers choose to adopt all three AMATs in Chongqing, our analysis shows that the most comprehensive combination is not the best choice for income. The adoption of the most comprehensive combination will bring about some inefficiency; in particular, excessive chemical input will not only bring about low efficiency but also have a negative impact on the external environment. The results show that agricultural technology extension agents should encourage tobacco farmers to make appropriate choices regarding technology combinations by fully considering the characteristics of their respective plots, further improving the input‒output efficiency of AMATs. For farmers in developing countries, adopting the appropriate technology combination will not only save their limited resources but also maximize the use of limited resources. The above analysis verifies the validity of Hypothesis 3.

## Section 6: Conclusions and implications

### Conclusions

An increasing number of farmers in developing countries participate in contract agriculture, which promotes the adoption of various advanced agricultural technologies and the development of standardized production. Although research on the effects of adopting various agricultural technology combinations on household welfare is increasing, there is little research on adopting additional combinations of technologies other than those used in standardized production. In China, due to the differences in resource endowment and natural environment, farmers often adopt one or more AMATs based on the standardized technical system required by the company to maximize their benefits. This study contributes to the literature in this area by examining the determinants and impacts of three AMATs and their combinations on incomes by using microscopic survey data from 346 households in Chongqing, China. We use a METE model to correct the selection bias and endogeneity due to observed and unobserved heterogeneity.

Our empirical results show that some factors affect tobacco farmers’ adoption of combinations of AMATs. Specifically, the household head’s education level, farming experience, flat land ratio, distance to extension station, and leased land ratio are found to affect the adoption of the three types of AMATs. The further away from the township a farmer is, the stronger the willingness of that tobacco farmer to adopt a combination of P_1_F_1_S_0_ is. The more technical training there is, the more significant the willingness of tobacco farmers to adopt the combination of P_0_F_1_S_1_ is. This indicates that the more times they participate in training, the more farmers can reduce their blindness to technical investment to a certain extent. Training can help tobacco farmers better understand and master advanced technologies.

With regard to the effect of adopting AMATs on outcomes, our findings suggest that estimates of outcome variables can be subject to sample selection bias when unobserved features are not considered. Our findings further reveal that there are some inefficiencies regarding the behavior of Chongqing tobacco farmers toward adopting AMATs. Taking the use of the three AMATs as a reference category, the use of additional combinations of P_1_F_1_S_0_ or P_0_F_1_S_1_ has a significant positive effect on income, and P_0_F_1_S_1_ has a larger positive effect. In addition, the adoption of P_1_F_1_S_0_ contributes to income growth mainly through the yield-enhancing effect, which has a significant positive impact on yield. The adoption of P_0_F_1_S_1_ mainly promotes income growth by the quality-enhancement effect, which has a significant positive impact on prices.

### Policy implications

Based on the conclusions presented above, we propose some constructive and operable policies. First, it is necessary for tobacco farmers to adopt AMATs; thus, tobacco farmers should be encouraged to choose appropriate combinations based on the conditions of their plots and crop growth on the basis of implementing standardized technical system. Second, because of the professionalism of AMATs, tobacco farmers cannot grasp them well. The company should make use of its professional advantages to conduct soil testing and analysis to help tobacco farmers adopt the right portfolio of AMATs. Third, based on the current behavior of Chongqing tobacco farmers, appropriately increasing the combination of fertilizers and soil improvement not only has a maximum income effect but also helps to protect the environment. The government should introduce some appropriate policies, such as technical subsidies and providing technical training and other policies, to encourage and guide tobacco farmers to adopt a combination of organic fertilizer and soil improvement and to reduce the use of additional chemical inputs such as pesticides. These policies could enormously improve tobacco farmers’ productivity and promote the sustainable development of tobacco planting in Chongqing.

## Supporting information

S1 File (RAR)
